# Smoking in Hospital Wards

**Published:** 1920-04-17

**Authors:** 


### Smoking in Hospital Wards

The note in The Hospital of April 3 with regard to the experiment at Cardiff, whereby patients in King Edward VII. Hospital are now allowed, tentatively, to smoke in the wards, has brought us the following statements. It will be noticed in which direction the evidence points. But we are ready to publish further experiences:

### The Experiences of Those Who Have Tried It.

For some time past the male patients of the Royal Albert Edward Infirmary, Wigan, have been allowed to smoke from 5 to 7 p.m. daily. The suggestion was duly considered before being adopted, and it has proved a comfort to many who would otherwise have been entirely cut off from a habit not easily or contentedly broken.

Further, the risk of fire is not greater than during the past five years, when nearly every civil hospital contained a certain number of wounded soldiers, who were invariably allowed to smoke practically all day. Also when smoking is forbidden in the wards some patients will elude the strictest vigilance on occasion and attempt to smoke under the bed-clothes, a far more dangerous proceeding than open smoking in given hours.

### The Practice in Scotland.

An institutional correspondent writes: I think that smoking is allowed at certain hours at the Edinburgh Royal Infirmary, and I believe that you will find that it is permitted elsewhere in Scotland.

### A Hundred Years' Experience.

The patients in the "Dreadnought" have been allowed to smoke in bed and in the wards for a hundred years. There are regulations, of course, and no smoking when the staff is going round or when dressing is being done or house men in the wards, but patients smoke at most other hours. Smoking is also allowed in the other hospitals and in the convalescent home of the Seamen's Hospital Society.

